# Estimating Allee Dynamics before They Can Be Observed: Polar Bears as a Case Study

**DOI:** 10.1371/journal.pone.0085410

**Published:** 2014-01-10

**Authors:** Péter K. Molnár, Mark A. Lewis, Andrew E. Derocher

**Affiliations:** 1 Department of Ecology and Evolutionary Biology, Princeton University, Princeton, New Jersey, United States of America; 2 Centre for Mathematical Biology, Department of Mathematical and Statistical Sciences, University of Alberta, Edmonton, Alberta, Canada; 3 Department of Biological Sciences, University of Alberta, Edmonton, Alberta, Canada; Bangor University, United Kingdom

## Abstract

Allee effects are an important component in the population dynamics of numerous species. Accounting for these Allee effects in population viability analyses generally requires estimates of low-density population growth rates, but such data are unavailable for most species and particularly difficult to obtain for large mammals. Here, we present a mechanistic modeling framework that allows estimating the expected low-density growth rates under a mate-finding Allee effect before the Allee effect occurs or can be observed. The approach relies on representing the mechanisms causing the Allee effect in a process-based model, which can be parameterized and validated from data on the mechanisms rather than data on population growth. We illustrate the approach using polar bears (*Ursus maritimus*), and estimate their expected low-density growth by linking a mating dynamics model to a matrix projection model. The Allee threshold, defined as the population density below which growth becomes negative, is shown to depend on age-structure, sex ratio, and the life history parameters determining reproduction and survival. The Allee threshold is thus both density- and frequency-dependent. Sensitivity analyses of the Allee threshold show that different combinations of the parameters determining reproduction and survival can lead to differing Allee thresholds, even if these differing combinations imply the same stable-stage population growth rate. The approach further shows how mate-limitation can induce long transient dynamics, even in populations that eventually grow to carrying capacity. Applying the models to the overharvested low-density polar bear population of Viscount Melville Sound, Canada, shows that a mate-finding Allee effect is a plausible mechanism for slow recovery of this population. Our approach is generalizable to any mating system and life cycle, and could aid proactive management and conservation strategies, for example, by providing *a priori* estimates of minimum conservation targets for rare species or minimum eradication targets for pests and invasive species.

## Introduction

For many animals and plants, the per capita population growth rate is positively correlated with population size or density when populations are small or sparse [1]. This phenomenon, known as a demographic Allee effect, is increasingly recognized as a population dynamics mechanism of key significance in applied ecology. In pest and invasive species control, for example, Allee effects may facilitate management and help species eradications [2], [3]. Conversely, the presence of an Allee effect may complicate the conservation of rare species, impede the success of reintroductions, and affect the development of sustainable harvesting strategies [4]–[8].

In general, demographic Allee effects can be categorized into strong and weak Allee effects, respectively [1], [9]. Strong Allee effects are characterized by the presence of an Allee threshold, defined as the critical population size or density below which the per capita growth rate becomes negative. Weak Allee effects, by contrast, still exhibit the positive relation between per capita growth and population size or density, but an Allee threshold is absent so that the per capita growth rate remains positive even at extremely low densities [1], [9]. The strength of an Allee effect influences the probability of extinction at low densities [1], making it crucial to determine accurate estimates of low-density growth rates for population management strategies. Knowledge of these growth rates can, for example, help define eradication targets for pests and invasive species, or minimum conservation and reintroduction targets for conservation management [1], [3], [10]–[12]. Similarly, Allee effects need to be accounted for in the harvest of exploited populations to avoid overly optimistic assessments of population resilience; neglecting an Allee effect, or accounting for it but with inaccurate low-density growth rate estimates, could inadvertently lead to the reduction of populations to sizes from which recovery is difficult [1], [4].

The determination of low-density growth rates, however, is difficult due to the low detection probability of individuals in low-density populations [13], [14]. Furthermore, many species may currently occur at high enough densities to be unaffected by the negative impacts of an Allee effect [15]. In such populations, direct measurement of low-density population growth to quantify the demographic consequences of potential Allee effects is impossible by definition. In smaller or immobile organisms, Allee effects can sometimes be demonstrated in laboratory or field experiments [16]–[18], but for large free-ranging animals, and in particular for threatened or endangered species, such population manipulation is usually unfeasible. Nevertheless, just because an Allee effect is unobservable, or just because an Allee effect does not affect a species' dynamics at current densities, does not mean that Allee effects can be ignored in population viability and other risk analyses [19]. If the impacts of an Allee effect on low-density growth cannot be quantified empirically, then explicit consideration of the mechanisms that may cause an Allee effect within process-based mathematical models can provide a first estimate of these quantities [15], [20], [21].

For a process-based approach, it is useful to differentiate between component and demographic Allee effects *sensu* Stephens *et al.* [22]. A component Allee effect refers to a positive relationship between any component of individual fitness and population density or size; a demographic Allee effect refers to a positive density-dependence of the per capita population growth rate. Mechanistically, Allee effects operate at the level of component Allee effects, which may or may not translate into demographic Allee effects, depending on the relative strengths of negative and positive density-dependence in different fitness components [22]. The mechanisms that could cause a component Allee effect differ between species, and may operate on demographic (e.g., reproduction, survival) or genetic (e.g., inbreeding, genetic drift) fitness components [1], [9]. Typical examples include, but are not limited to, a reduced likelihood of finding mates at low densities (e.g., butterflies, Atlantic cod *Gadus morhua*), reduced efficiency of broadcast spawning (e.g., sea urchins), reduced success of anti-predator behavior (e.g., meerkat *Suricata suricatta*), reduced foraging efficiency (e.g., black-browed albatross *Thalassarche melanophrys*), or genetic Allee effects (e.g., Florida panther *Puma concolor coryi*); see [1], [9] for reviews.

The first step in using process-based models for evaluating the risk of Allee effects requires determining the likely mechanisms leading to a component Allee effect [9]. Second, these mechanisms need to be formulated in a process-based model, which – due to its focus on the mechanisms causing the Allee effect – can often be parameterized and validated from data on the relevant mechanisms, even if an Allee effect has not been observed [15], [20], [23]. Finally, the component Allee effect model must be linked to a population dynamics model to evaluate whether and how a component Allee effect may translate into a demographic Allee effect [1], [9], [21]. Here, we illustrate this approach for polar bears (*Ursus maritimus*), where a mate-finding component Allee effect was identified as a potential conservation concern, but it remains unclear if this component Allee effect could lead to a demographic Allee effect [15], [20].

Polar bears are solitary, non-territorial animals [24]. Low population densities and a dynamic sea ice habitat result in an unpredictable spatial distribution of mates and low frequencies of mate encounters [25]. Mate-finding thus becomes a key factor in polar bear reproduction, rendering this species vulnerable to mate-finding Allee effects [15]. Additionally, due to a prolonged and highly sex-selective harvest, male numbers have been reduced in most Canadian polar bear populations, raising concerns that males might become so depleted that many females would become unable to find a mate [26], [27]. To address these concerns, Molnár *et al.* [15] developed a process-based model for the polar bear mating system that accounts for mate searching, and the formation and dissolution of breeding pairs. Their model was parameterized using observed frequencies of solitary males, solitary females, and breeding pairs during the mating season, and described these mating dynamics data well. The model predicts the proportion of fertilized females as a function of population density and operational sex ratio, and showed that female mating success depends nonlinearly on the operational sex ratio in such a manner that a sudden reproductive collapse could occur if males are severely depleted (cf. Fig. 4 in [15]). Moreover, the authors demonstrated an interaction between the operational sex ratio and population density, where high-density populations require relatively fewer males per female than low-density populations to ensure high female mating success. Applying the model to the population of Lancaster Sound, Canada, Molnár *et al.* [15] concluded that this particular population was large enough not to warrant concerns regarding mate-finding Allee effects, despite a highly female-biased sex ratio. Similar conclusions likely apply to other high-density populations, although climate-change-induced declines in reproduction, survival, and population density, may eventually render some of these populations vulnerable to Allee effects [20], [28], [29]. Low-density populations with strongly biased sex ratios, by contrast, may already be experiencing reduced population growth due to mate-finding limitations, but this cannot be evaluated with the mating model of Molnár *et al.* [15] alone, as this model does not consider the population dynamics consequences of reduced mating success.

Here, we link the within-year mating dynamics of polar bears, described by the model of Molnár *et al.* [15], to their between-year population dynamics, using a matrix projection model tailored to the life history of polar bears. This approach is used to explore whether and how a mate-finding component Allee effect may translate into a demographic Allee effect, and aims to provide a process-based predictive framework for mate-finding Allee effects in polar bears. The model predicts fertilization probabilities and resultant population growth rates as a function of population density, age- and sex-structure. In particular, it enables an estimate of the Allee threshold, even though an Allee effect has not been documented in polar bears to date. The framework could aid conservation and harvest managers in precautionary risk analyses by accounting for mate-limitation. For illustration, we apply our model to the Viscount Melville Sound population where historic overharvest [30] resulted in extremely low population numbers with few adult males remaining, and population recovery appears slow. Our findings show that, for reasonable estimates for the range of model parameters found in Viscount Melville Sound, a mate-finding Allee effect is a plausible mechanism for the slow recovery.

## Methods

### Model Development

Polar bears are long-lived, reach sexual maturity when about five years old, and usually live for at least twenty, and in some cases up to 25–30, years [24]. The mating season lasts from late-March/early-April to late-May/early-June, when males seek females by following their tracks on the sea ice [31]. Upon encounter and with the female accepting the male, a breeding pair is formed that remains together 1–4 weeks [24], [32]. The mating system is characterized by males locating, defending, and fertilizing females one after another [33]. The number of females a male can fertilize is restricted by population density, mate-searching efficiency, pair association length, and mating season length [15]. A female's mating success, defined as the probability of being fertilized in a given mating season, is determined both by population density and operational sex ratio (the number of sexually mature males relative to the number of mature females that are available to mate, that is, unaccompanied by cubs-of-the-year or yearlings [15]). As such, mating success can be characterized as both density- and frequency-dependent. After mating, blastocyst implantation is delayed until autumn when pregnant females enter dens and give birth to 1–3 cubs [34], [35]. For the next 2.5 years, cubs rely on maternal care for survival and growth, and will usually die if the mother dies during this period [36]. At 2.5 years of age, the cubs are weaned and the mother again becomes available for mating.

**Figure 1:**
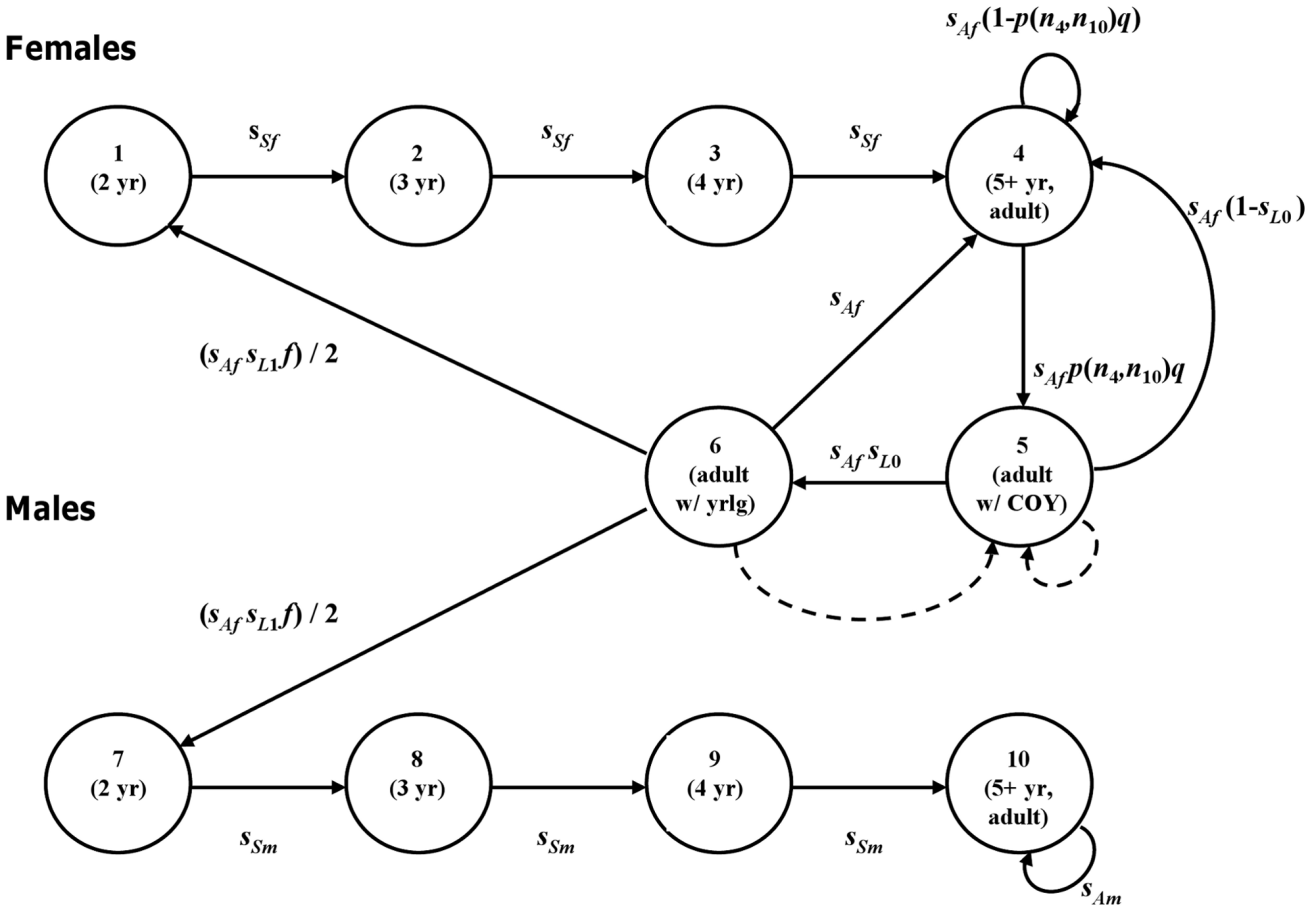
The polar bear life cycle underlying the two-sex matrix projection model with maternal care, *A_n_*. Stages 1–6 are females, stages 7–10 are males. *s_Sf_*, *s_Af_*, *s_Sm_* and *s_Am_* are the probabilities of subadult and adult survival from one mating season to the next for females and males, respectively; *s_L_*_0_ and *s_L_*_1_ are the probabilities of at least one member of a cub-of-the-year (COY) or yearling (yrlg) litter surviving from one mating season to the next; *f* is the mean number of 2-year-olds in a litter that survives to this age. A 1:1 sex ratio in dependent offspring is assumed. *p*(*n*_4_,*n*_10_) is the probability that an adult female that is not accompanied by dependent offspring is fertilized, given the numbers of such females (*n*_4_) and adult males (*n*_10_). *q* is the conditional probability, given survival, that a fertilized female will produce at least one COY that survives to the following mating season. Dashed arrows indicate transitions that are theoretically possible but infrequent, and are thus omitted from the population model for simplicity. The figure is modified from [28], [37].

To understand under what conditions mate limitation would lead to a demographic Allee effect, we link the within-year mating dynamics of polar bears to their between-year population dynamics. For the between-year dynamics, we represent the polar bear life cycle in the stage-structured matrix model of Hunter *et al.* [28], , summarized here for convenience (Fig. 1). This model tracks both female and male numbers over time, stratified by reproductive status and age:
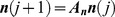
(1)


Here, the population vector ***n***(*j*) represents the number of individuals in each stage at time step *j*, with the entries *n*_1_, *n*_2_ and *n*_3_ corresponding to subadult (nonreproductive) females aged 2, 3, and 4 years, *n*_4_ to adult females (≥5 years) available for mating, *n*_5_ to adult females accompanied by one or more cubs-of-the-year, and *n*_6_ to females with yearling cubs. The male segment of the population is tracked in (*n*_7_,…,*n*_10_), with *n*_7_, *n*_8_ and *n*_9_ corresponding to subadult males aged 2, 3, and 4 years, respectively, and *n*_10_ representing sexually mature adult males (≥5 years). The matrix ***A_n_*** projects ***n***(*j*) from the end of one mating season to the beginning of the next and is defined as
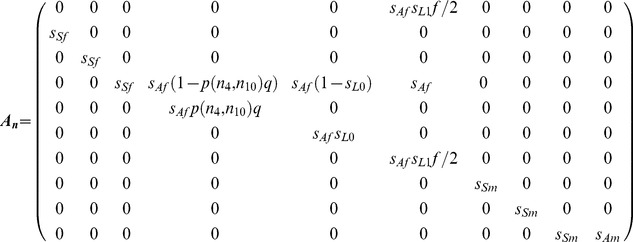
(2)


The parameters *s_Sf_*, *s_Af_*, s*_Sm_* and *s_Am_* represent the probabilities of subadult and adult survival for females and males, respectively, from the mating season in year *j* to the mating season in year *j*+1. Transition from stage 4 (adult females without dependent offspring) to stage 5 (adult females with cubs-of-the-year) depends on the probability of finding a mate, *p*, and the conditional probability *q* of a successful pregnancy given successful mating, that is, the probability that at least one cub is born and survives to the next mating season. Because stages 5 (adult females with cubs-of-the-year) and 6 (adult females with yearlings) are modeled as mother-cub units, transition between these two stages depends on the survival of the mother (*s_Af_*) and the probability that at least one member of a cub-of-the-year litter survives to the next year (i.e., the cub-of-the-year litter survival probability), *s_L_*_0_. Similarly, fecundity (the link from stage 6 to stages 1 and 7; Fig. 1) includes the survival probability of the mother (*s_Af_*), the probability that at least one member of a yearling litter survives to the following year (i.e., the yearling litter survival probability), *s_L_*_1_, and the parameter *f*, representing the mean number of 2-year-olds in a litter of this age. The parameters *s_L_*_0_, *s_L_*_1_, and *f* are calculated from the lower-level parameters *s_C_* (annual survival probability of an individual cub-of-the-year), *s_Y_* (annual survival probability of an individual yearling), and *c*_1_ and *c*_2_ (probabilities of having one or two cubs, respectively, in case of a successful pregnancy), using the formulae in Appendix B of [28]. For these calculations, a 1:1 sex ratio and sex-independent survival for cubs-of-the-year and yearlings (*s_C_*, *s_Y_*) are assumed throughout. Transitions from stages 5 or 6 into stage 5 (Fig. 1: dashed lines) occur rarely, do not influence the population dynamics significantly [37], and are omitted from the model for simplicity. For a detailed discussion of this model, see [28], [37].

Our matrix model differs from Hunter *et al.*'s [28], [37] in that the probability of finding a mate, *p*(*n*_4_,*n*_10_), depends on the density of females and males that are available for mating at the beginning of each mating season *j* [15]. This approach accounts for inhibited mate-finding in low-density populations, introduces nonlinearity into the projection matrix ***A_n_***, and may give rise to a demographic Allee effect when too few males or females are present. To include this in the population projections, we use Molnár *et al.*'s [15] mating model (summarized below) to update *p*(*n*_4_,*n*_10_) in ***A_n_*** annually with the estimated proportion of available females that are fertilized, based on the number of females and males available for mating at the beginning of the mating season, *n*_4_ and *n*_10_:
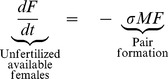
(3a)

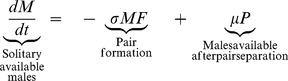
(3b)

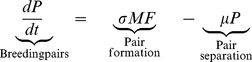
(3c)Here, *F*(t), *M*(t), and *P*(*t*), represent the respective densities of solitary unfertilized females, solitary males searching for mates, and breeding pairs, during the mating season. The left-hand sides of equations 3a-c represent the respective rates of change in these quantities, which depend on the rates of pair formation and pair separation. Pair formation is modeled using the law of mass action (with pairs formed at rate *σ*), which captures the processes of mate searching in polar bears, and was shown to describe the observed mating dynamics well [15]. After pair formation, breeding pairs stay together for *μ* ^−1^ time units, thus separating at rate *μ*. For simplicity, the model further assumes that all mortality losses to the population occur outside the mating season (thus subsumed in the survival parameters of the projection matrix ***A_n_***). The mating dynamics model (3) is run for the length of the mating season, which begins each year at the start of the projection interval *j*−>*j*+1 (denoted *t* = 0) and lasts *T* time units. In each year *j*, the model is initialized with the number of females and males available for mating at the beginning of the mating season, scaled to the habitat area *H*: *F*(0) = *n*_4_(*j*)/*H*, *M*(0) = *n*_10_(*j*)/*H*, and *P*(0) = 0. The probability of fertilization in year *j* is given by *p*(*n*_4_(*j*),*n*_10_(*j*))  = 1-*F*(*T*)/*F*(0), which is obtained by numerically integrating equation (3) from *t* = 0 to *t* = *T* [15].

To explore which conditions may lead to a demographic Allee effect due to mate limitation, we systematically initialize the combined mating/population dynamics model with all possible combinations of male and female densities, and evaluate the resultant population growth rate for each case. Although we acknowledge that stochasticity may substantially influence the dynamics of low-density populations, we keep our simulations deterministic to illustrate the direct impacts of the Allee effect on population growth. As such, we put particular emphasis on determining whether polar bears are likely to exhibit strong or weak Allee dynamics, and estimate the likely Allee threshold by determining which initial conditions lead to population persistence or extirpation, respectively. Because the initial population age/stage-structure may also influence model outcomes, and specifically whether a population persists or becomes extirpated ([38]–[40], cf. also Results), we consider three different initial age/stage-structures in our simulations: (i) an “old” population where all females and males are sexually mature adults (i.e., in stages 4 and 10) at the beginning of the projection, (ii) a “young” population where all bears are 2-year-old subadults (i.e., in stages 1 and 7) in the beginning, and (iii) an “intermediate” population, where females and males are distributed between age classes (stages 1–4 for females, and 7–10 for males) according to proportions that would be obtained under a stable-stage distribution with no mate limitation (i.e., with p = 1). In all scenarios, adult females are without dependent offspring at the beginning of the projection (i.e., stages 5 and 6 are empty), so that the Allee threshold can be determined without the obscuring effects of past reproduction. Throughout, we do not include negative density-dependence in the matrix ***A_n_*** as we are primarily interested in the dynamics of low-density populations.

### Model Parameterization

Initially, we parameterize the projection matrix ***A_n_*** with a generic parameter set (Table 1) that can be regarded as representative of a “typical” polar bear population [27]. Moreover, this specific parameter set was previously used to explore the sex-structured population dynamics of polar bears without mate limitation [27], making our results directly comparable to this earlier study. Specifically, we set the survival probabilities of subadult females and males *s_Sf_* = *s_Sm_* = 0.95, and of adults *s_Af_* = *s_Am_* = 0.96 [27]. The probability of successful pregnancy given fertilization was set at *q* = 0.725 [29], and the probabilities of having one or two cubs-of-the-year with successful pregnancy were *c*_1_ = 0.2 and *c*_2_ = 0.8, respectively, yielding a mean litter size of 1.8 in stage 5 [27]. The survival probabilities of individual cubs and yearlings were set *s_C_* = 0.72 and *s_Y_* = 0.77, respectively [27]. Together, these parameters imply litter survival probabilities of *s_L_*_0_ = 0.88 for cub-of-the-year litters and *s_L_*_1_ = 0.85 for yearling litters. The resulting mean number of 2-year-olds in a litter of this age is *f* = 1.327. Without mate limitation (i.e., assuming *p*(*n*_4_,*n*_10_)≡1), these parameters yield a stable-stage growth rate of *λ* = 1.056. The within-year mating dynamics parameters *σ* (pair formation rate), *μ* ^−1^ (mean pair association length) and *T* (mating season length) were set as determined in [15] (i.e., *σ* = 49.2 km^2^ d^−1^, *μ* ^−1^ = 17.5 days, *T* = 60 days).

**Table 1:** Parameter definitions for the polar bear projection model given by equations 1–2 (between-year population dynamics) and equation 3 (within-year mating dynamics), with estimates given for a generic polar bear population and the Viscount Melville Sound population.

		Generic Population	Viscount Melville Sound
**Between-Year Population Dynamics**			
*s_C_*	Annual survival probability of an individual cub-of-the-year	0.720	0.571*
*s_Y_*	Annual survival probability of an individual yearling	0.770	0.941*
*s_Sf_*	Annual survival probability of subadult females	0.950	0.957
*s_Sm_*	Annual survival probability of subadult males	0.950	0.924
*s_Af_*	Annual survival probability of adult females	0.960	0.957
*s_Am_*	Annual survival probability of adult males	0.960	0.924
*q*	Probability of successful pregnancy given successful fertilization	0.725	1.000
*c*_1_	Probability of having one cub in case of successful pregnancy&	0.200	0.360
*c*_2_	Probability of having two cubs in case of successful pregnancy&	0.800	0.640
*s_L_*_0_	Probability that at least one member of a mean cub-of-the-year litter survives to the next year§	0.881	0.728
*s_L_*_1_	Probability that at least one member of a mean yearling litter survives to the next year§	0.853	0.957
*f*	Mean number of 2-year-olds in a litter that survives to this age§	1.327	1.265
**Within-Year Mating Dynamics**			
*σ*	Pair formation rate [km^2^ d^−1^]	20.6	20.6
*μ* ^−1^	Mean length of pair association [d]	17.5	17.5
*T*	Mating season length [d]	60	60
*H*	Habitat area [km^2^]	100,000	104,540%

Parameter estimates for the between-year population dynamics are from [27] for the generic population, and from [30] for the Viscount Melville population; estimates for the within-year mating dynamics are from [15].

These probabilities did not differ significantly between males and females in the study population [30]; the sex-specific estimates of [30] were therefore averaged between males and females.

^&^ Calculated from the mean litter sizes (*LS*) reported in the source studies, using *c*_1_+2*c*_2_ = *LS* and *c*_1_+*c*_2_ = 1 (i.e., assuming triplet litters do not occur).

*s_L_*_0_, *s_L_*_1_ and *f* were calculated from *s_C_*, *s_Y_*, *c*_1_, and *c*_2_ using the formulae in Appendix B of [28].

Habitat area corresponding to the total marine area within the population boundaries; three scenarios were considered, with mating bears concentrating in 15.5%, 50% or 100% of this habitat area, respectively (cf. text for details).

Each of these parameters may take slightly different values in different polar bear populations and/or may be altered by climate change. Thus, we explored the sensitivity of the Allee threshold to the model parameters by varying them one at a time, and repeating the simulations outlined above. For this, we reduced each parameter of the projection matrix ***A_n_*** such that the stable-stage population growth rate *λ* was reduced by 50% from its baseline towards *λ* = 1 (i.e., from *λ* = 1.056 to *λ* = 1.028), yielding *s_C_* = 0.424, *s_Y_* = 0.490, *s_Sf_* = *s_Sm_* = 0.817, *s_Af_* = *s_Am_* = 0.929, *q* = 0.318, *c*_1_ = 0.902 and *c*_2_ = 0.098. This scaling was employed to allow for a common baseline of comparison between the different life history parameters, and specifically, to separate the direct effects of each parameter on the Allee threshold from the effects each parameter would have on this threshold via its effects on the population growth rate *λ*. The above life history parameters will likely decrease with climate change but it is unclear whether and in which direction the parameters of the mating model would change [20], [41]. Thus, we explored the sensitivity of the Allee threshold to these parameters in both directions, increasing and decreasing the pair formation rate *σ* by ±50%, and increasing and decreasing the pair association length *μ* ^−1^ by ±7 days, relative to their baseline values.

### Application to the Viscount Melville Sound population

The Viscount Melville Sound population is shared between the Northwest Territories and Nunavut, Canada, and is located in the ocean channel separating Melville Island to the north, and Banks and Victoria islands to the south; see [30], [42] for a detailed population description. The population boundaries were established using mark-recapture movement data [43], DNA analysis [44], and cluster analysis of radio-telemetry data [45], [46], indicating that this population is demographically closed [30]. In the second half of the 20th century, the population was overharvested for several decades, resulting in a population decline from 500–600 bears to 161±34.5 bears in the last census in 1989–1992 [30]. Moreover, because the harvest was strongly sex-biased, the male-to-female ratio among adult bears declined from 0.96 during the mid-1970s to 0.41 during the last census [30]. Since the census, harvest management has been aimed at population recovery. Initially, a five-year harvest moratorium was implemented, followed by a harvest that was thought to be sustainable [30], [42]. The current status of the population is unclear, but a new assessment is ongoing.

Here, we used our models to explore whether, and to what degree, the recovery of the Viscount Melville population may have been hindered or slowed since the last census by a demographic Allee effect due to mate limitation. For this, we applied the matrix projection model (1), coupled with the mating model (3), as outlined above. The matrix ***A_n_*** was parameterized using the vital rate estimates reported in [30], which yield a population growth rate *λ* = 1.059 in the absence of mate limitation and harvest (Table 1). The mating model parameters were set as above (Table 1). The models were initialized with 161 bears distributed between the stage classes of the population vector ***n*** according to the standing age-/reproductive-stage distribution in 1989–1992. Because the mating model requires densities as input, we transformed these numbers into densities by scaling them to the habitat area that is used during the mating season, *H*. For this, we considered three scenarios because the mating season distribution is poorly documented. First, we used the mating model to estimate the degree of mating season aggregation that would be required to obtain the litter production rate observed in 1989–1992. For this, we averaged the age-specific litter production rates reported in [30] according to the standing stage distribution of the population and assumed no unsuccessful pregnancies in fertilized females (*q* = 1), obtaining a fertilization probability *p* = 0.845 for 1989–1992. Given the numbers of males and females, this value of *p* implies that mating bears were extremely aggregated to about 15.5% of the marine area of the population, or *H* = 16,238 km^2^. The implied density was about three times higher than the mean density reported for Canadian populations [43], so we contrasted this high-density scenario against an intermediate-density scenario with *H* = 52,270 km^2^, and a low-density scenario with *H* = 104,540 km^2^ (corresponding to polar bears utilizing 50% or 100% of the available marine area during the mating season). For each of these scenarios, we projected the population forward from the last census year (1992). We report the expected population trajectories for the cases of (i) no harvest to illustrate the maximum potential for population growth given the initial conditions, and (ii) documented harvest [42] included to the present and harvest continued into the future using current quotas.

## Results

**Figure 2:**
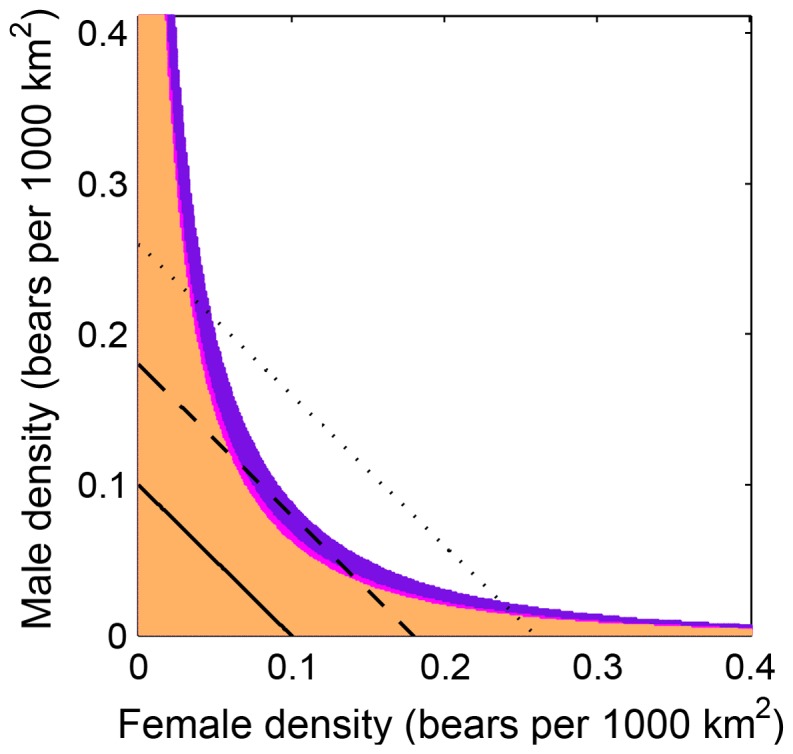
The mate-finding Allee threshold in polar bears. Three initial age-structures are considered, corresponding to an “old”, an “intermediate” and a “young” population. In the old population, all females and males are sexually mature adults (i.e., in stages 4 and 10, cf. Fig. 1) at the beginning of the projection, whereas in the young population all bears are 2-year-old subadults (i.e., in stages 1 and 7). In the intermediate population, females and males are distributed between age classes (stages 1 to 4 for females, and 7–10 for males) according to proportions that would be obtained under a stable-stage distribution. In all scenarios, adult females are taken to be without dependent offspring at the beginning of the projection. Scenarios marked orange lead to extirpation for all three initial age-structures; scenarios marked pink lead to extirpation in the young and intermediate population, but not in the old population; purple marks scenarios that lead to extirpation in the young population only. The solid, dashed, and dotted lines correspond to polar bear populations of fixed densities 0.10, 0.18 and 0.26 bears per 1000 km^2^, respectively, illustrating (i) that a population will always become extirpated at extremely low densities regardless of sex ratio or age-structure (solid line), (ii) that at somewhat higher densities a population may or may not persist at balanced sex ratios depending on its age-structure, but always becomes extirpated with biased sex ratios (dashed line), and (iii) that at even higher densities a population is always expected to persist regardless of age-structure, unless the sex ratio is extremely biased (dotted line).

Our models suggest that the component Allee effect of reduced fertilization probability under low population densities outlined in [15] leads to a strong demographic Allee effect in low-density polar bear populations (Fig. 2). The Allee threshold separating conditions that lead to population persistence or extirpation, respectively, hereby depends on male and female density, as well as the initial age-structure of the population. Populations at extremely low densities were always extirpated regardless of the initial operational sex ratio or age-structure (Fig. 2: solid line). Populations at somewhat higher densities, by contrast, may or may not persist – depending on their initial age-structure – at approximately balanced sex ratios, but always become extirpated with biased sex ratios (Fig. 2: dashed line). This dependence of the Allee threshold on age-structure is observed because in younger populations some immature individuals die before reproducing, implying that younger populations require a higher density for persistence than older populations (contrast the three initial age-structures in Fig. 2). At intermediate to high densities, populations are expected to persist regardless of age-structure, unless the initial operational sex ratio is extremely biased (Fig. 2: dotted line). Nevertheless, even in persisting populations (i.e., those above the Allee threshold), mate-finding limitations can reduce population growth over several generations; it is only in medium- to high-density populations that the restrictions imposed by mate searching become negligible (Fig. S1). The Allee threshold is slightly asymmetrical between the sexes, because a stronger sex ratio skew is permissible towards females than males for population persistence. For example, in the “old” population scenario with a total density of 0.18 bears per 1000 km^2^, the population requires at least 0.059 females per 1000 km^2^ (implying 0.121 males per 1000 km^2^), but only 0.035 males per 1000 km^2^ (implying 0.145 females per 1000 km^2^) (Fig. 2: dashed line).

**Figure 3:**
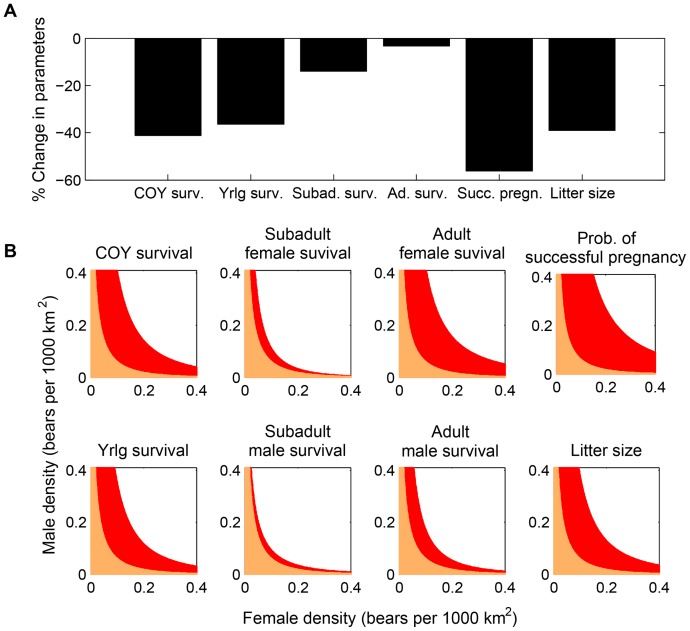
The dependence of the Allee threshold on the parameters of the projection matrix *A_n_*. To allow for a common baseline of comparison between these life history parameters, we reduced parameters one at a time such that the stable-stage population growth rate *λ* was reduced by 50% from its baseline (i.e., from *λ* = 1.056 to *λ* = 1.028). This scaling ensures that the direct effects of each parameter on the Allee threshold are separated from the effects each parameters would have on this threshold via its effects on the population growth rate *λ*. Panel (A) shows the proportional amount by which each parameter needed to be reduced relative to its baseline value to obtain *λ* = 1.028. (B) Scenarios marked orange (replotted from Fig. 2) would lead to extirpation both with the baseline parameters and the reduced parameters; scenarios marked red would lead to extirpation with the reduced parameters, but not with the baseline parameters. Each panel considers the “old” population scenario, where all females and males are sexually mature adults (i.e., in stages 4 and 10, cf. Fig. 1) at the beginning of the projection, and no female is accompanied by dependent offspring.

The location of the Allee threshold is also sensitive to the life history parameters encompassed in the projection matrix ***A_n_***. As expected, a decrease in any of the survival or reproduction parameters moves the threshold to higher male and female densities (Fig. 3B), whereas an increase in these parameters moves the threshold to lower densities (not shown). Separating out the effects of each life history parameter on the Allee threshold from their effects on the stable-stage population growth rate *λ* (cf. Methods) reveals high sensitivity of the Allee threshold to adult female survival and all reproduction parameters (cub-of-the-year survival, yearling survival, probability of successful pregnancy, litter size), moderate sensitivity to adult male survival, and low sensitivity to subadult survival (Fig. 3B). These results contrast with the sensitivity of the stable-stage growth rate *λ*, which is highly sensitive to adult survival, moderately sensitive to subadult survival, and relatively insensitive to the reproduction parameters ([37], [47], cf. also Fig. 3A). Furthermore, these sensitivity analyses illustrate how different combinations of the reproduction and survival parameters of the projection matrix ***A_n_*** could lead to differing Allee thresholds, even if they imply the same asymptotic stable-stage population growth rate *λ*. For the mating dynamics parameters, the Allee threshold is sensitive to pair formation rate (higher rates implying a lower threshold) but insensitive to pair association length (Fig. S2), in accordance with the sensitivity of the fertilization probability *p*(*n*_4_,*n*_10_) to these two parameters [15].

**Figure 4:**
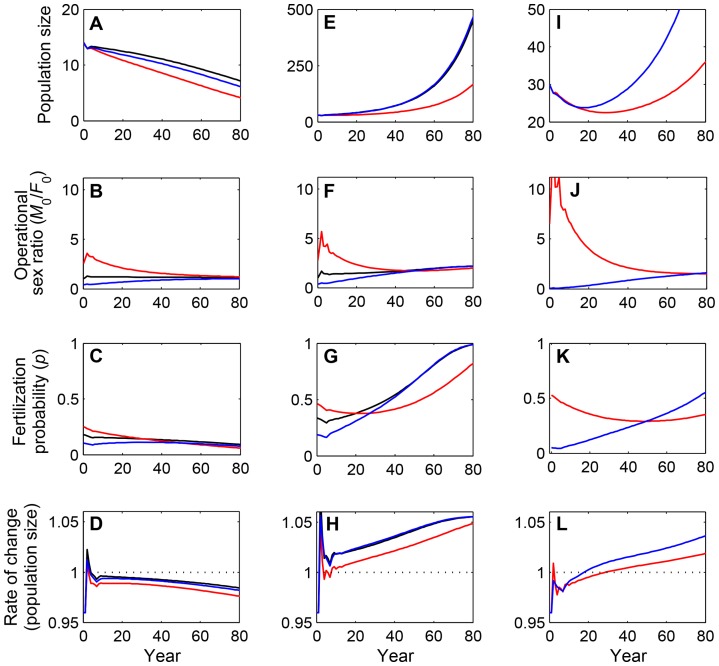
Transient population growth, operational sex ratios and fertilization probabilities for populations near the Allee threshold. Each simulation is initialized with an “old” population, where all females (*F*_0_) and males (*M*_0_) are sexually mature adults at the beginning of the projection, and no female is accompanied by dependent offspring. Populations with male-biased, balanced, and female-biased sex ratios are shown in red, black, and blue, respectively. Population sizes in panels (A,E,I) correspond to independent adults and subadults only, that is, they do not include dependent offspring. (A–D) Three populations where mate limitation leads to extirpation (red: *M*_0_ = 10, *F*_0_ = 4; black: *M*_0_ = 7, *F*_0_ = 7; blue: *M*_0_ = 4, *F*_0_ = 10); (E–H) Three populations that increase to persistence even though initial population growth is slowed by mate limitation (red: *M*_0_ = 22, *F*_0_ = 8; black: *M*_0_ = 15, *F*_0_ = 15; blue: *M*_0_ = 8, *F*_0_ = 22); (I–L) Two populations that initially show a long decline before eventually increasing to persistence (red: *M*_0_ = 26, *F*_0_ = 4; blue: *M*_0_ = 2, *F*_0_ = 28). For all populations, the habitat area is set as *H* = 100,000 km^2^.

The growth trajectory of polar bear populations are determined by the initial male and female densities as well as the initial age-structure. Below the Allee threshold, populations decline to extirpation as discussed above, but these declines are slow without demographic stochasticity due to the long generation time of polar bears (Fig. 4A). The rates of decline vary over time and are influenced by changes in the operational sex ratio and population density. Of these, the operational sex ratio converges to a relatively balanced ratio over time (Fig. 4B), and this may somewhat slow the decline (Fig. S1). The probability of fertilization, however, still decreases with declining densities (Fig 4C), thus leading to an overall acceleration of the population decline rate (Fig. 4D). Above the Allee threshold, populations persist but the growth of low-density populations is initially still limited by mate scarcity (Fig. 4E–L). Depending on the initial age- and sex-structure, fertilization probability may remain well below one for many generations (Fig. 4G,K), so that the impacts of the mate-finding Allee effect are observed for prolonged periods (Fig. 4H,L). Furthermore, for populations with extremely biased sex ratios that are just above the Allee threshold, it is possible that the population would initially show a prolonged decline before beginning to increase (Fig. 4I). For example, the “old” population scenario, initiated with 26 males and 4 females in 100,000 km^2^ of habitat, declines for ∼30 years before increasing, whereas with 2 males and 28 females, the initial decline lasts ∼20 years (Fig. 4I,L). The reasons for these patterns differ between the male- and female-biased scenarios. For the male-biased sex ratio, the decline occurs despite relatively high fertilization rates (Fig. 4K, red line), and is due to a lack of females producing offspring (Fig. 4J: red line). For the female-biased sex ratio, the initial decline is due to extremely low fertilization probabilities (Fig. 4K: blue line). In both cases, the initial decline is reversed when enough offspring have matured to supplement the breeding pool (Fig. 4I,L). Therefore, such populations may overcome the mate-finding Allee effect due to their long generation time, but inbreeding may result. Generally, population growth is maximized with an approximately balanced operational sex ratio at low densities, and with a female-biased sex ratio at intermediate to high densities (Fig. S1; also contrast black, blue and red lines in Fig. 4).

**Figure 5:**
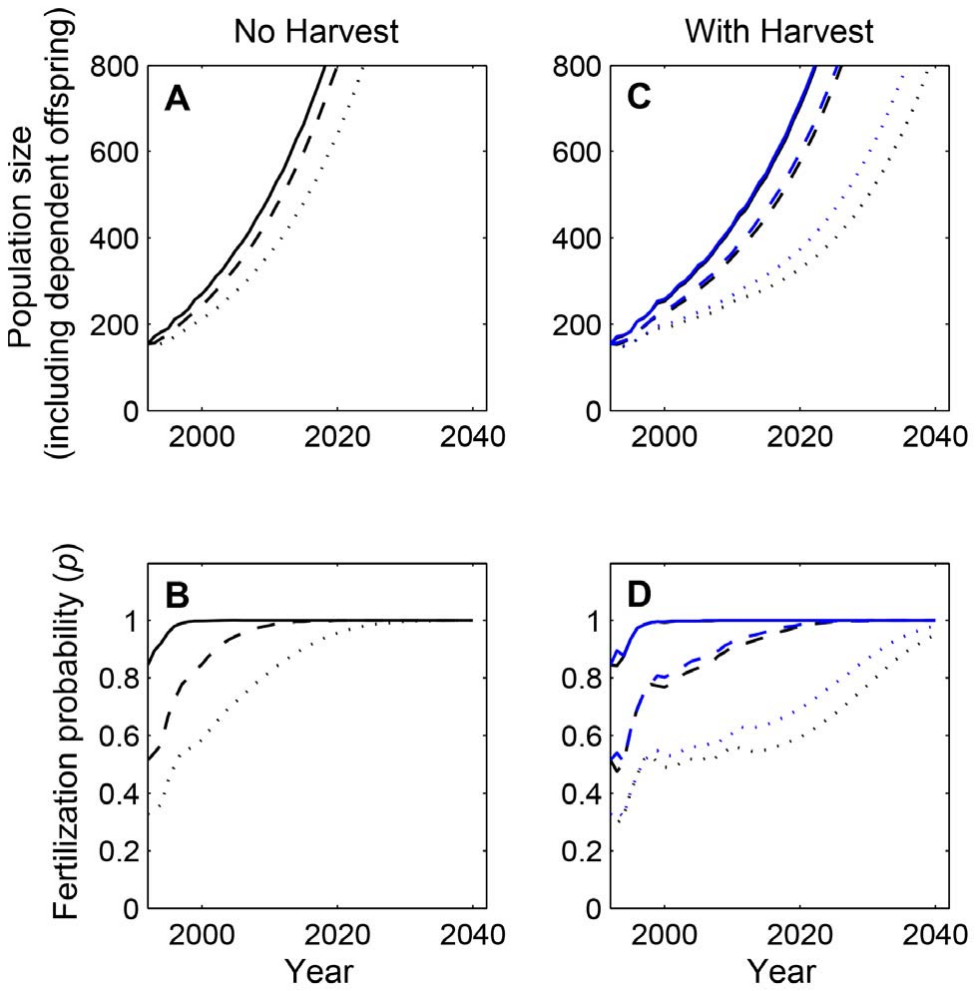
Population projections and corresponding fertilization probabilities for the Viscount Melville Sound population. All projections begin at the last day of the latest population census (1989–1992), and are initialized using the reported standing age/reproductive-stage distribution. Models are parameterized using the life history parameters reported in [41], and the mating dynamics parameters of [15]. (A,B) Population projections for the (hypothetical) case of no harvest illustrating the maximum potential for population growth given the initial conditions in 1992, (C,D) population projections with the actual harvest rates included to the present and harvest continued into the future using current quotas. For both the “no-harvest” and “harvest” scenarios, we consider (i) a low-density case where polar bears are spread out through the entire available habitat within the population boundaries (dotted lines), (ii) an intermediate-density case where bears are concentrated in half of the available habitat (dashed lines), and (iii) a high density (mating aggregation) case where bears concentrate in ∼15.5% of the available habitat area (solid lines). For the “harvest”-scenario, two projections are shown for each density case, either assuming that all harvest occurs directly before (black) or directly after (blue) the mating season.

When applied to Viscount Melville Sound, our model illustrates the necessity of determining the mating season distribution of polar bears for population projections. Under the high-density scenario, that is, if bears continued to aggregate each mating season since 1992, the model suggests the population should have recovered to nearly historical levels. Fertilization probabilities would have reached unity near the year 2000, and – assuming that the vital rate estimates of 1992 remained unchanged and unaffected by negative density-dependence at high densities – by 2013 the population would have increased to ∼500 bears despite ongoing harvest (Fig. 5C,D: solid lines). Furthermore, if the harvest had been completely discontinued since 1992, the population could have even increased to ∼590 bears by 2013 (Fig. 5A: solid line). These projections contrast with those for the intermediate- and low-density scenarios, where the absence of mating aggregations leads to reduced fertilization probabilities and more conservative projections of population growth. In these cases, the population would have remained below its maximum growth potential for two to three decades even without harvest (Fig. 5A,B: dashed and dotted lines), and with harvest, this effect would have been exaggerated further (Fig. 5C,D): With intermediate densities, fertilization probabilities would remain below unity until about 2020, and the population would only have been expected to increase from 161±34.5 bears in 1992 to 406–422 bears in 2013 (Fig. 5C,D: dashed lines). With low densities, the effects of mate-limitation become even more pronounced, with an expected population increase to only 269–295 bears by 2013, and the mate-finding Allee effect not fully overcome until mid-century (Fig. 5C,D: dotted lines). In each of these density scenarios, harvesting after the mating season gives slightly more optimistic projections than harvesting before the mating season, as this allows for slightly increased fertilization probabilities of females (Fig. 5C,D: contrast black and blue lines).

## Discussion

### The a priori estimation of Allee Dynamics

Allee effects have garnered much attention in ecology and conservation biology, and numerous theoretical models have been developed for exploring their causes and consequences. Parameterization and application of these models generally requires estimating the influence of Allee effects on low-density population growth rates, but such estimates are often unavailable. Due to this lack of data, population viability analyses often do not include the possibility of an Allee effect [48]–[50]. As outlined earlier, this omission could have severe consequences, for example, in the management of endangered or exploited species.

Here, we have argued that the *a priori* estimation of low-density population growth rates and the Allee threshold is possible – even if low-density growth data are unavailable – by considering the mechanisms causing the Allee effect within process-based models. The advantages of this approach are threefold. First, unlike in population models that use a phenomenological Allee effect term [51], no *a priori* assumption about the existence of an Allee effect needs to be made. In polar bears, the component Allee effect of mate-limitation arose naturally from the mating dynamics, and the demographic Allee effect arose as a consequence of evaluating resultant fertilization probabilities in conjunction with all other demographic parameters. Second, an *a priori* assumption about the form of the Allee effect is also unnecessary. Indeed, our approach revealed that population density, sex ratio, age-structure and vital rates all interact non-linearly to determine the Allee threshold in polar bears. This result stands in contrast to most existing Allee effect models which often make the simplifying assumption that mating success is solely determined by density or sex ratio [51], [52]. Third, our approach enables estimates of low-density growth rates and the Allee threshold, even if a population is currently at a high enough density to be unaffected by a mate-finding Allee effect. This is possible because our model focuses on the limitations imposed by mate-searching and other mating system characteristics. As such, mechanistic mating models, and by extension demographic models that account for potential mate limitations, require additional data to those traditionally used in population viability analyses. The parameters of our mating model, for example, can be estimated from the observed pairing dynamics [15], male-female encounter rates [53], or movement patterns [54], [55].

Population viability analyses in general, and matrix models in particular, aim to predict future population sizes from the underlying demographic parameters, reproduction and survival [56]. Our approach complements this by predicting reproduction from the mechanisms determining mating success. The framework is flexible and easily adapted to other species, because the mating model can be modified to any mating system (e.g., [15], [23], [57]) and matrix models can be used to represent any life cycle [56]. Indeed, similar modeling approaches to that taken here – linking the within-year mating dynamics to the between-year population dynamics – were previously used to estimate Allee thresholds and assess the efficiency of pest control strategies in gypsy moth (*Lymantria dispar*) [58], and to understand the temporal and spatial dynamics of a house finch (*Carpodacus mexicanus*) invasion [57]. Whether or not a demographic Allee effect will arise in a given species depends on the specifics of their mating dynamics and their life cycle, which need to be accounted for in model formulations. In harem-breeding ungulates, for example, mate-searching limitations are negligible, but limitations in the capacity of males to inseminate females exist [59], [60]. In such a system, operational sex ratio will influence population growth but density will not [59], [60], as would also be predicted by an appropriate modification of the mating model we employed [15].

Mechanistic approaches to estimating Allee thresholds are not limited to mate-finding Allee effects. Courchamp *et al.* [61] and Rasmussen *et al.* [62], for example, used bioenergetic considerations to determine the lower critical size of African wild dog (*Lycaon pictus*) packs, and their results could inform population viability analyses in a manner similar to that outlined here. Ultimately, Allee effects can arise for numerous reasons, and need to be included in population viability analyses. A lack of low-density population growth data to parameterize population models should not be a reason to disregard Allee effects in risk analyses if data on the underlying mechanisms can be obtained.

### The risk of mate-finding Allee effects in polar bears

Population viability analyses are commonly used in polar bear management, for example, to assess conservation status, to evaluate potential impacts of climate change, or to determine harvest quotas (e.g. [28], [30]). Such analyses have traditionally been based on the matrix model of Hunter *et al.* [28], [37], or on the population simulation program RISKMAN [63]. While RISKMAN is an individual-based model, its structure is similar to Hunter *et al.*'s model, in particular regarding its ability to account for the three-year reproductive cycle of polar bears. Neither model incorporates interactions between males and females, so that all polar bear viability analyses to date have implicitly assumed that female mating success is independent of male density. This is, for example, reflected in the recommended harvest ratio of two males for every female in Canadian populations – a ratio that was determined from RISKMAN simulations that aimed for maximizing yield while only requiring that a relatively arbitrary proportion of the male population (as defined through abundance and mean age, but not density) was maintained [27]. Because mate-finding Allee effects were not considered in these assessments, the validity of these recommendations is questionable, especially for low-density populations and populations with strongly female-biased sex ratios.

Our models emphasize the necessity to consider mate-finding Allee effects in polar bear risk analyses, and provide a means for estimating low-density population growth rates and the Allee threshold before an Allee effect can be observed. While the interaction between density, sex ratio, age-structure, and the rates of reproduction and survival, makes it impossible to provide an analytic formula for the Allee threshold, this is unnecessary as our models are easily implemented numerically. Simulations can thus be tailored to the idiosyncrasies of different populations, allowing population-specific estimates of low-density growth, the Allee threshold, and age- and sex-structured harvest quotas. Such risk analyses should also account for climate-change-induced effects on reproduction and survival, as observed and predicted declines in these parameters (e.g., [20], [29], [64]–[66]) could interact with mate-finding limitations. Not only are low-density populations more likely to experience mate scarcity and thus reduced growth (Fig. S1), but reductions in the demographic parameters of the matrix ***A_n_*** could move the Allee threshold itself (Fig. 3). It is therefore theoretically possible that a population that is currently above the Allee threshold would fall below the threshold with reduced reproduction and survival, implying a switch from positive to negative population growth. Accounting for future changes in reproduction and survival due to predicted changes in sea ice again faces the data limitations of yet unobserved conditions, thus also requiring a process-based modeling approach [20]. Here, energy budget models can be used for predicting changes in reproduction and survival that result from changes in energy uptake and utilization [20], [29], [67], and movement models can predict the impacts of an altered sea ice configuration on mate-finding [20], [41]. Ultimately, population viability analyses rely on survival and reproduction estimates, which are often assumed to remain stable for the projection period [30], [68]. This simplifying assumption is unlikely to hold true in many systems, and may lead to unrealistic assessments of risk [69]. Mechanistic models focusing on the extrinsic (e.g., impacts of sea ice condition on energy budgets) or intrinsic (e.g., impacts of density and stage-structure on mate-finding) processes determining reproduction and survival could outline how these parameters are likely to change over time. We advocate these approaches as complementary to existing viability models, especially to avoid overly optimistic harvest quotas.

For population viabilities analyses, the mating season distribution of males and females emerged as a novel parameter of interest, as illustrated by our analyses of the Viscount Melville Sound population. Depending on this distribution, a variety of population trajectories were possible here, ranging from exponential growth with nearly certain female fertilization under the high-density scenario, to substantially reduced growth with mate-finding limitations under the lowest-density scenario. While a full population viability analysis is beyond the scope of this paper, it seems clear that the risk of extirpation would be increased in the latter case. The sustainability of the current harvest quota should thus be reassessed in light of these possibilities, especially because it cannot be determined from currently available data which density scenario reflects the population distribution. The relatively high litter production estimates reported in [30] imply mating aggregations during the early 1990s, which would suggest rapid population recovery from overharvesting. However, preliminary data from the ongoing population assessment suggests that little recovery has occurred during the last 20 years (A. Derocher, unpublished data), and this might be indicative of currently low mating season densities and mate-finding limitations. These two views on the past and present mating season distributions are not necessarily contradictory, for example, because climate change may have rendered a larger habitat area suitable for this population [41], thereby reducing mating densities from those in the early 1990s.

In general, we view our analyses of the Viscount Melville Sound population as illustrative of the role Allee effects may play in polar bears, and of the implications these effects could have for management. In addition to emphasizing the uncertainty that results from the lack of distribution data, we emphasize the need to collect data on male-female encounter rates, mate choice, and female mating success to improve the accuracy of the mating model. Female mating success is almost exclusively determined by the pair formation rate parameter *σ*, which can also be viewed as the encounter rate between males and females multiplied by the probability that a female accepts a male upon encounter (i.e., the degree of mate choice) [15]. Here, we have assumed that the rate of pair formation equals the rate observed in Lancaster Sound (a high-density population) [15], but it is also possible that mate choice varies adaptively with density and operational sex ratio [70], or that movement rates (and thus encounter rates) vary between populations due to differences in sea ice configuration [71]. Such population-specific idiosyncrasies could influence the pair formation rate *σ*, and thus low-density growth and the Allee threshold (Fig. S2). Until data on the factors influencing pair formation become available, this uncertainty should be accounted for in viability analyses. For management applications, our models will also need to be extended to include demographic and environmental stochasticity, as random mortality events and random fluctuations in the population sex ratio could substantially influence mating success, population growth rates and extirpation risk, especially at low densities [72]. It is noteworthy that with such stochasticity, a population may go extinct even when above the Allee threshold (or persist despite being below the Allee threshold) with some probability [1], [73]. It would be straightforward to merge our approach with existing viability analysis frameworks as our models not only predict the Allee threshold, but indeed the population growth rate for any density and population composition (Fig. S1).

For monitoring, our simulations suggest that the standard interval between polar bear population surveys (15 years in Canada) is inadequate for low-density populations and populations with strongly biased sex ratios. With infrequent assessments, dynamic changes in mating success and other demographic parameters may go unnoticed, and such changes could impact population viability, especially if harvest is continued based on outdated information. Moreover, our analyses caution that the eventual fate of low-density populations may not be immediately apparent from the observed population size trajectory. With long generation times, transient dynamics may be long, and declines or increases may be slow (Fig. 4). While some indication of the likely direction of growth could be obtained from changes in age-structure, sex ratio, and fertilization rates (Fig. 4), it seems that in such cases a precautionary approach to harvesting is the only justified strategy. That a mate-finding Allee effect has not been documented in polar bears should not be taken as indication that Allee effects can be ignored in viability analyses; Allee effects are hard to observe, and may have been missed because fertilization rates are not routinely monitored, time series of population growth do not exist, and/or because all polar bear populations may have so far occurred at high enough densities to remain unaffected by mate scarcity. The consequences of disregarding Allee effects in the management of low-density populations may, however, be severe.

## Supporting Information

Figure S1
**The impact of mate limitation on short-term population growth.** The geometric mean population growth rate 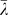 is shown for two projection intervals (15 years and 45 years) as a function of initial population density and operational sex ratio. Each panel considers the “old” population scenario, where all females and males are sexually mature adults (i.e., in stages 4 and 10, cf. Fig. 1) at the beginning of the projection, and no female is accompanied by dependent offspring. The operational sex ratio yielding maximal population growth as a function of initial population density is shown by a thick black line for each projection interval. Note that (i) population growth is reduced over a wide range of densities and sex ratios relative to the population growth that would be obtained under a stable-stage distribution with no mate limitation (*λ* = 1.056), (ii) for a given density and sex ratio, the population growth rate depends on the time period considered due to initial transient dynamics and the impacts of positive density-dependence, (iii) population growth is maximized with an approximately balanced operational sex ratio at low densities, and with a female-biased sex ratio at intermediate to high densities (black lines), (iv) the initial operational sex ratio that maximizes population growth by the end of the projection interval depends on the length of the considered projection (contrast black lines between the two panels), and that (v) the contour line corresponding to no change in population size (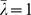) approximates but does not exactly correspond to the Allee threshold shown in Fig. 2 due to the effects of initial (and potentially long, cf. Fig. 4I–L) transient dynamics.(EPS)

Figure S2 **The dependence of the Allee threshold on the mating model parameters** ***σ*** **and** ***μ*** **^−1^.** For both pair formation rate (*σ*) and pair association length (*μ* ^−1^), we contrasted outcomes obtained with the baseline parameter values *σ* = 49.2 km^2^ d^−1^ and *μ* ^−1^ = 17.5 d against outcomes obtained with parameter values corresponding to more (*σ* increased by 50% or *μ* ^−1^ decreased by 7 days) and less (*σ* decreased by 50% or *μ* ^−1^ increased by 7 days) efficient mating, respectively. In both panels, scenarios marked red lead to extirpation for all considered parameter values; scenarios marked orange lead to extirpation with the baseline parameter values and the values corresponding to inefficient mating, but not with efficient mating; blue marks scenarios that lead to extirpation with inefficient mating only. The three scenarios largely overlap in panel (B), illustrating the insensitivity of the Allee thresholds to the pair association length *μ* ^−1^. Both panels consider the “old” population scenario, where all females and males are sexually mature adults (i.e., in stages 4 and 10, cf. Fig. 1) at the beginning of the projection, and no female is accompanied by dependent offspring.
(EPS)
